# New-Onset Pain and Neurologic Deficit After Intraoperatively Uneventful Lumbar Spine Surgery: A Prospective Observational Case Series and Proposed Management Algorithm

**DOI:** 10.7759/cureus.113900

**Published:** 2026-08-03

**Authors:** Ravi Ranjan Rai, Bharatkumar R Dave, Mikeson Panthackel, Denish Patel, Ajay Krishnan, Shivanand C Mayi, Mirant B Dave, Arjit Vashishtha, Amritesh Singh, Saurabh S Kulkarni, Yogenkumar Adodariya, Kishan Panjwani

**Affiliations:** 1 Spine Surgery, Stavya Spine Hospital and Research Institute, Ahmedabad, IND; 2 Spine Surgery, Bhavnagar Institute of Medical Science (BIMS), Bhavnagar, IND; 3 Orthopedics, Mahatma Gandhi Medical College and Research Institute, Aurangabad, IND

**Keywords:** lumbar spine surgery complications, new onset deficits, new onset pain, postoperative deficits, postoperative pain, spine complications

## Abstract

Background

Postoperative pain and neurological deficit after lumbar fusion surgery are uncommon but noted and are often under-reported, which may have financial and medico-legal consequences.

Objective

To investigate and identify the likely causes of new-onset symptoms after lumbar spine surgery.

Method

This is a single-center, prospective study carried out from November 2017 to April 2020. Among the patients undergoing lumbar spine surgeries for stenosis or degenerative spondylolisthesis during the study period, all those who presented with new-onset pain or neurological deficit within the first month were included in the study. Patients with pre-existing infection, fracture, tumor, previous surgery, intraoperative events such as dural tear or root injury, and intraoperatively detected screw breach were excluded. All these patients underwent detailed clinical and radiological evaluation to ascertain the causative factor, and treatment was initiated accordingly.

Results

A total of 2497 surgeries were performed during the study period, among which 31 patients satisfied the inclusion criteria and were enrolled in the study. Of these, 12 patients developed neurological deficit, while 19 had only radicular pain. The etiology was identified in 14 patients in the form of screw breach, epidural hematoma, infection, fascicle herniation, recurrent disc prolapse, foraminal stenosis, and implant-related causes. The etiology could not be established in 17 patients. Conservative treatment was successful in the majority of patients; only four required surgery.

Conclusion

New-onset pain or neurological deficit after lumbar spine surgery, although rare, is a known complication. In spite of detailed clinical and radiological evaluation, the cause of neurological deficit or pain could not be confirmed in most cases.

## Introduction

Historically, lumbar spine surgery carried a reputation for variable outcomes and a high susceptibility to complications. Today, however, advancements in imaging technology, a deeper understanding of spine biomechanics, and the evolution of surgical techniques have led to improved clinical expectations. Despite these advances, the development of new symptoms following an otherwise uncomplicated lumbar procedure remains a formidable clinical challenge. Current literature indicates that the incidence of iatrogenic neurological deficits following lumbar spine surgery ranges from 0.8% to 6.1%, with many cases attributable to specific surgical approaches or instrumentation [1].

Traditionally, unexpected or suboptimal outcomes after spine surgery have been grouped under the highly nonspecific umbrella term “Failed Back Surgery Syndrome” (FBSS) [2-3]. Critically, FBSS fails to differentiate between symptoms persisting from the pre-operative period, natural disease progression, and entirely new symptoms arising in the immediate post-operative window. While recent literature has introduced “immediate Failed Back Surgery Syndrome” (iFBSS) to more precisely describe early symptom deterioration following discectomy, there remains a pressing need for standardized terminology that strictly isolates newly developed radicular or neurological issues across a broader spectrum of lumbar procedures [4].

To provide greater clinical clarity, we introduce the term “New-Onset Pain or Deficit” (NOPD) to specifically characterize radicular symptoms and neurological deficits observed in the immediate post-operative period, within one month of surgery. NOPD is defined as the emergence of a neurological deficit that was absent during pre-operative evaluation, or the presentation of severe radicular symptoms (Visual Analog Scale (VAS) > 6) in the contralateral limb or a completely new dermatome, severe enough to warrant an extended hospital stay or require re-admission. When encountered, NOPD not only compromises patient outcomes but also carries substantial financial and medico-legal implications.

Therefore, the primary objective of this study is to systematically investigate the underlying etiologies of NOPD. The secondary objective is to propose a structured management algorithm to guide its timely diagnosis and treatment. It should be noted that this study is not designed to establish the exact incidence of complications following lumbar spine surgery, but rather to evaluate the causes of NOPD when it occurs and devise a practical treatment pathway.

## Materials and methods

This is a single-centre, prospective study carried out from November 2017 to April 2020. After institutional review board approval (CTRI No. 2017/11/010343), cases were prospectively enrolled. All patients aged 18 to 80 years undergoing lumbar spine surgery for stenosis or degenerative spondylolisthesis, who presented with NOPD within one month of surgery, with all records available and a minimum of one-year follow-up available, were included in the study. NOPD was defined as the development of a neurological deficit that was not observed in the pre-operative evaluation or a complaint of radicular pain (VAS > 6) along a different dermatome than pre-op, which was severe enough to warrant an extended hospital stay or required re-admission. Patients aged less than 18 or more than 80 years, and those operated on for trauma, tumour, or pre-existing infection, were excluded from the study. Also, patients with known intra-operative events such as dural tear, root injury, or screw breach were excluded from the study, since the likely cause of NOPD was known in these cases and did not require investigation.

**Figure 1:**
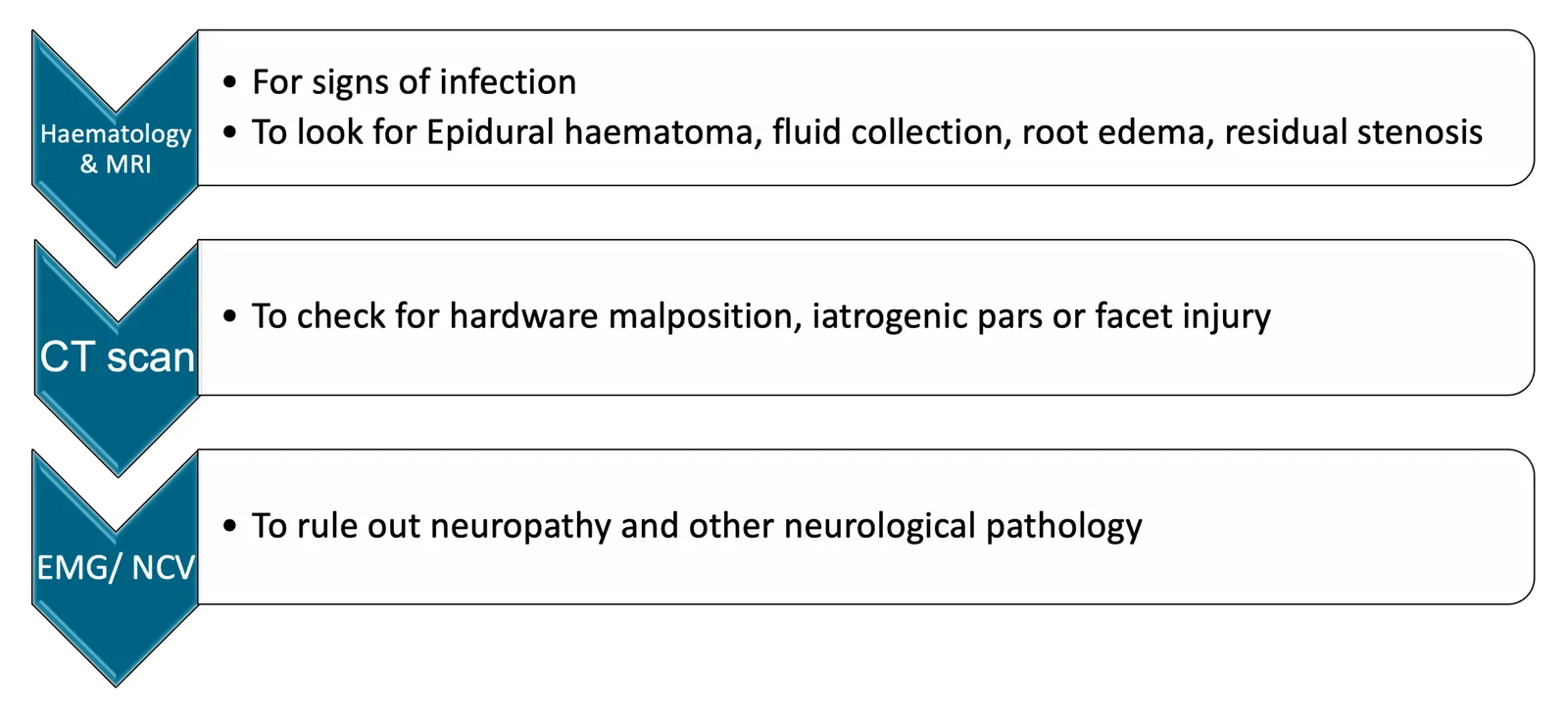
Algorithm for the evaluation of NOPD after lumbar spine surgery. NOPD: New Onset Pain or Deficit; EMG: Electromyography; NCV: Nerve conduction velocity.

All patients enrolled underwent detailed history-taking and clinical evaluation, followed by blood investigations. Subsequent evaluation was guided by the algorithm detailed in Figure 1.

Whenever a patient presented with NOPD, blood tests and MRI were performed on the same day or as soon as possible. When an initial finding correlated with the patient’s clinical presentation, targeted treatment was initiated, and symptom resolution was monitored. However, if the pain persisted or worsened, the remaining investigations in the sequence were carried out until the definitive underlying cause was identified. Patients were either treated symptomatically or with correction of the underlying cause, when identified. All patients were followed up for a minimum period of 12 months.

Treatment strategies for NOPD encompassed both conservative and surgical management. Conservative management followed a standardized institutional protocol consisting of bed rest and a three-day pharmacological regimen. This regimen included IV diclofenac 75 mg twice daily, or IV tramadol thrice daily if diclofenac was contraindicated, oral pregabalin 75 mg twice daily, and IV pantoprazole 40 mg once daily. Patients who demonstrated a positive clinical response, defined as a reduction in the VAS score to below 6, were transitioned to equivalent oral medications and subsequently discharged. In confirmed cases of surgical site infection (SSI), culture-directed IV or oral antibiotics were administered based on specific sensitivity reports.

Surgical decision-making was highly individualized. Interventions depended on the specific identified etiology, the mechanical feasibility of surgical correction, the severity of the neurological deficit, an inadequate response to the conservative treatment trial, and informed patient consent. For example, non-compressive etiologies such as battered root syndrome were strictly managed conservatively, whereas distinct mechanical complications, such as a major screw breach, warranted prompt surgical revision. Ultimately, the decision to proceed with surgical re-exploration was complex; it relied on shared decision-making between the patient and the surgical team and served as a clinical complement to the diagnostic algorithm.

Criteria used to identify the causes

Symptomatic Epidural Hematoma (SEH) 

Suspected in patients presenting with acute motor deficits (e.g., foot drop, quadriceps weakness) or severe focal pain. Diagnosis was confirmed via MRI demonstrating a fluid collection compressing the neural elements corresponding to the new clinical deficit.

Surgical Site Infection (SSI) 

Suspected in patients presenting with fever, localized swelling, wound discharge, or disproportionately increased radicular symptoms. Because immediate post-operative ESR and CRP are unreliably elevated, diagnosis relied on a continuously rising CRP level alongside clinical signs, confirmed by targeted deep tissue sampling for organism identification before empirical antibiotic therapy. At our institute, all patients had their baseline CRP value noted on the fourth postoperative day, and the value was rechecked if there was any NOPD, preferably after the seventh day, to allow good comparison. Any doubling of the CRP value in the second measurement compared with the first was considered suggestive of SSI.

Screw Breach

Any screw breach that was detected and redirected intraoperatively was excluded from the study, even if the patient subsequently developed post-operative pain corresponding to that corrected trajectory. Conversely, cases where a breach was missed intraoperatively but later identified during the diagnostic workup for new-onset pain were included. In these instances, diagnosis was confirmed using standard or 3D-reconstructed CT scans to accurately localize and grade the breach (e.g., medial breach at the exiting nerve root).

Foraminal or Residual Stenosis

Suspected when symptoms suggested suboptimal decompression. This was investigated and confirmed via post-operative MRI to identify residual compression in the neural foramen or lateral recesses.

Stretching of the Root

Considered in cases involving large interbody cage placement or significant deformity correction, such as correction of lumbar kyphosis with listhesis. Diagnosis was established radiographically by measuring and comparing the distance from the sciatic notch to the relevant neural foramen (e.g., L4) on both pre-operative and post-operative standing radiographs to quantify iatrogenic lengthening.

Battered Root Syndrome

Considered a diagnosis of exclusion, this was identified in patients presenting with acute, severe radicular symptoms, hours to days post-surgery, whose MRI revealed isolated ipsilateral nerve root edema without obvious mechanical compression.

Fascicle Herniation

Suspected in patients presenting with delayed deficits, such as delayed bladder retention or new dermatomal pain, following an apparently uneventful surgery. Diagnosis was definitively confirmed via surgical exploration identifying an undetected dural tear with extruding nerve fascicles.

Diabetic/Peripheral Neuropathy

Suspected in patients with a known history of diabetes where other mechanical causes of NOPD were ruled out. This was evaluated using electrophysiological studies, including electromyography and nerve conduction studies, to differentiate peripheral neuropathy from structural spinal pathology.

Statistical analysis

Data were collected prospectively and analyzed using descriptive statistics. Categorical variables, such as the identified etiologies of NOPD and the types of clinical presentation (pain vs. neurological deficit), are expressed as frequencies and percentages. The small sample size precludes inferential statistical analysis.

## Results

A total of 2497 lumbar spine surgeries were performed during the study period for degenerative pathology. Among these, 31 patients were found to have NOPD and satisfied the inclusion criteria for the study. Twelve patients were observed to have developed neurological deficit, while 19 patients had only radicular pain. Pain subsided in all except one patient by 3 to 6 months. Neurological deficit was observed to have fully or partially recovered to at least Grade 3 in 8/12 patients. The remaining four patients did not experience any recovery of motor power until the final follow-up.

The cause of NOPD was identified in 14 patients, while in 17 patients, the cause could not be identified in spite of all investigations (Table 1). Two patients required revision surgery, two patients underwent wound wash and evacuation of hematoma/collection, and one patient was managed with percutaneous drain insertion. The remaining patients were managed conservatively with symptomatic treatment.

**Table 1:** Causes of NOPD after lumbar spine surgery in our cohort. NOPD: New-onset pain or deficit.

Cause	Number of patients
Unidentified	17
Epidural haematoma	3
Surgical site infection	2
Screw breach	2
Stretching of the root	1
Foraminal stenosis	1
Epidural fibrosis	1
Battered root syndrome	1
Diabetic neuropathy	1
Dural tear	1
Fascicle herniation	1
Total number of patients with NOPD	31

**Figure 2:**
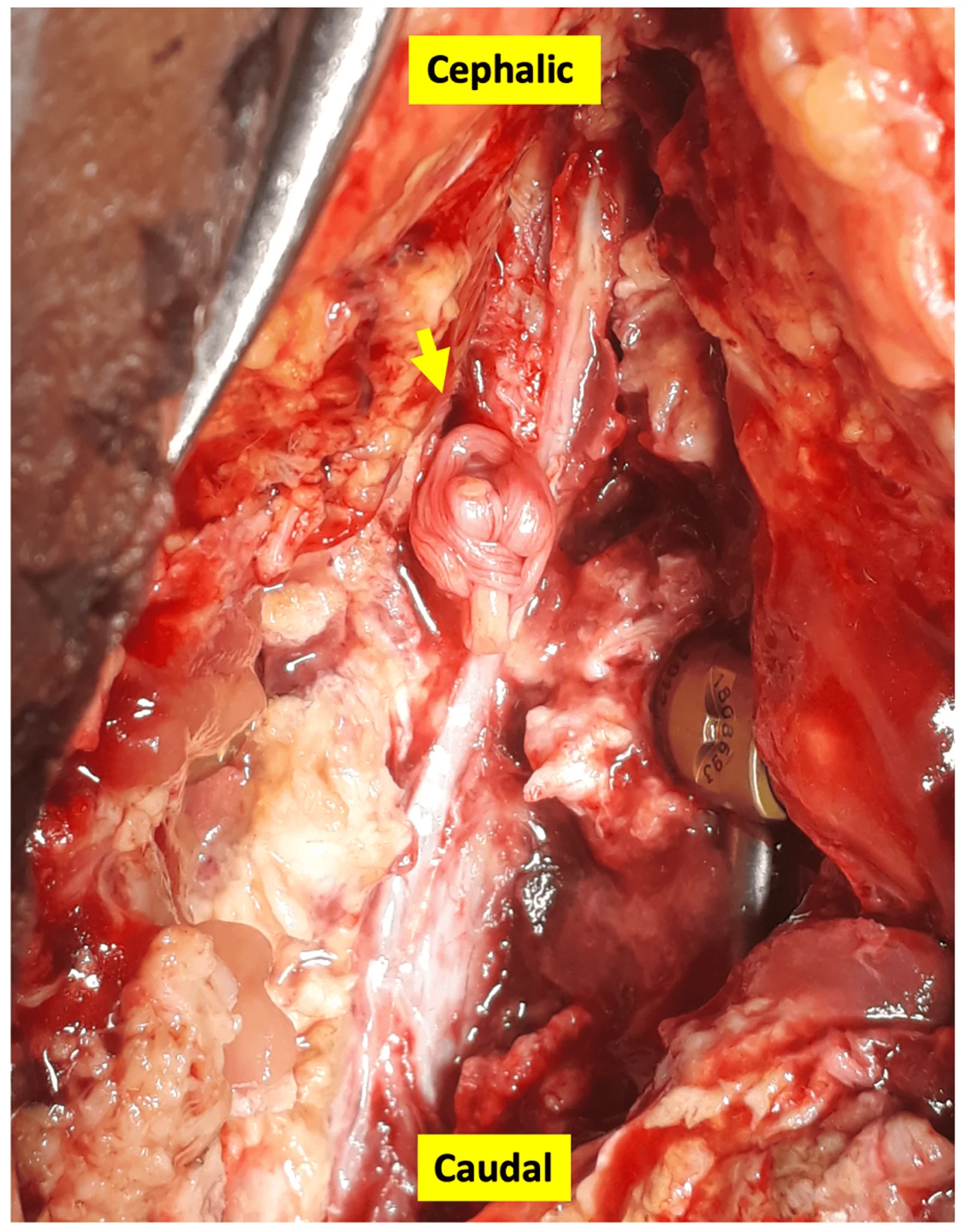
Intraoperative image of a 58-year-old female who underwent revision surgery for fascicle herniation. A 58-year-old female underwent L3-L4 and L4-L5 TLIF. One week postoperatively, she presented with NOPD, including bladder symptoms, and was taken for exploration. The yellow arrow shows an undetected dural tear with fascicle herniation. The tear was extended, the roots were repositioned, and the dural defect was repaired. TLIF: Transforaminal lumbar interbody fusion; NOPD: New-onset pain or deficit.

We had one case of undetected dural injury where the patient presented after one week with NOPD, including bladder retention. Subsequent MRI revealed a fluid collection at the index operative site. Upon exploration, we found fascicle herniation from the undetected dural injury. The dural defect was extended, the fascicles were put back, and the dural defect was repaired. The patient recovered to a functional level over a period of 6 weeks and had complete recovery at 6 months’ follow-up. The intraoperative image is shown in Figure 2.

## Discussion

Most studies in the literature on complications after lumbar spine surgery have focused on FBSS. Since the definition of FBSS is not specific, it is not helpful in describing or managing specific complications of surgery. With advancements in spine imaging and improved understanding of spine biomechanics, we should be able to identify and manage complications more efficiently than before. The present study particularly concentrates on investigating the causes of immediate postoperative complications and their management through a new term, NOPD. NOPD includes cases that develop neurological deficits or pain in the contralateral limb or along a different dermatome, which was not present before the surgery. The study is not designed or aimed to show the exact incidence of complications with lumbar spine surgery, but rather to investigate the possible causes of NOPD and devise a treatment algorithm for the same [4].

The nature of NOPD, as well as the duration of the symptom-free interval after surgery, gives some clues regarding the causative pathology [5]. In patients without any substantial improvement in presenting symptoms, the most likely causes are incorrect pre-surgical diagnosis, suboptimal decompression, psychological issues, or secondary gain. For patients presenting after a pain-free interval of weeks to months, the surgeon needs to consider recurrent disc herniation, battered root syndrome, or arachnoiditis. For patients presenting after an interval of months to years, the likely pathology would be recurrent disc herniation, recurrent spinal stenosis, or adjacent segment disease. In the present study, we included only those patients presenting with NOPD within one month of surgery. We broadly classified the causes of NOPD into mechanical causes, infective causes, and unidentified causes.

Mechanical causes

SEH, screw breach, foraminal stenosis, dural tear, and fascicle herniation comprised eight cases of NOPD in our study. These are cases where the proposed algorithm helps with early identification, and prompt corrective measures can improve surgical outcomes.

In the present series, we had three patients with NOPD diagnosed as SEH, of whom one developed quadriceps weakness, the second had left foot drop, and the third had only pain. The patient with quadriceps weakness underwent wound wash and resuturing under general anaesthesia, while the other two were managed conservatively. All three recovered to functional power by 3 months postoperatively. Preoperative diastolic blood pressure, intraoperative gel foam use, postoperative drain output, advanced age, use of anticoagulants, and long procedures are some of the identified risk factors for the development of SEH. Early diagnosis and surgical evacuation of haematoma are key to preventing neurological deterioration and improving clinical outcomes [6].

The present series had one patient with a medial screw breach at L5 who required screw revision. Another patient with Grade 1 screw breach was managed conservatively. Screw breaches are best identified on CT scan. With the advent of enabling technology and the availability of 3D CT-based navigation, complications related to screw breach have substantially reduced [7].

Suboptimal decompression, more commonly in the neural foramen and lateral recesses, is known to give rise to FBSS [5] and, in our series, was found to be responsible for NOPD. This may be due to unfamiliarity with the anatomy, inaccurate diagnosis, or suboptimal decompression due to fear of creating iatrogenic instability. In our experience, suboptimal decompression is directly related to the surgeon’s training and experience and is usually not expected in experienced hands. If in doubt, this can be easily confirmed on postoperative MRI scan.

Fascicle herniation through an undetected dural tear can result in postoperative neurological deficits, including bowel and bladder involvement. MRI typically reveals a fluid collection at the operative site. When symptomatic, these cases necessitate revision surgery to identify and repair the dural rent, allowing for the safe repositioning of the neural elements.

Infective causes

The incidence of SSI after lumbar spine surgery varies from 0.7% to 12%, leading to increased healthcare costs, patient morbidity, and mortality [8]. Fever, swelling, wound discharge, pain over the operative site, or increased radicular symptoms point toward the likelihood of SSI. Blood markers such as ESR and CRP may be elevated in the postoperative period and are therefore unreliable for diagnosing SSI in the immediate postoperative period. A rising CRP level may provide an early indication. A high index of suspicion is required to diagnose SSI early. The treatment of SSI depends on whether the infection is superficial or deep and on the duration since surgery. Thorough wound debridement is recommended for deep infections; however, there is literature suggesting that appropriate IV antibiotics started within the first 10 days may be effective [9]. Identifying the primary focus of infection and the organism is key to the successful treatment of SSI. Hence, every attempt should be made to take a sample before starting empirical antibiotics. We had two cases of SSI in our series: one patient with an evident collection on MRI developed quadriceps weakness and required debridement, and the other had new-onset pain only. Both healed well with appropriate antibiotics. The patient with quadriceps weakness recovered functional power by six weeks, and at one year, the clinical outcome was good in both cases.

Unidentified causes

Excessive bleeding during the procedure or aggressive root retraction may lead to battered root syndrome and may result in NOPD. This may be evidenced by root oedema on MRI. Identifying root oedema can be challenging on early postoperative MRI, and an experienced radiologist becomes invaluable here. It is usually transient, with symptoms seen a few hours to a few days after surgery, and settles with strong anti-inflammatory medications. In our experience, battered root syndrome is more commonly seen in a surgeon’s early career, and the incidence declines with the surgeon’s experience and technique in handling the nerve roots in tight stenosis.

Perineural scarring or epidural fibrosis occurs after most spinal decompressive procedures, but it is thought to be an extremely uncommon cause of clinical failure [10-12]. Factors suggested as contributors to scarring include excessive neural retraction, excessive bleeding, conjoined nerve roots, and the use of cottonoid patties. Perineural scarring may play a role in battered root syndrome, which typically presents with incomplete resolution of sciatica and increasing radicular symptoms over 3 to 6 months from the time of surgery.

Diabetic neuropathy is a known cause of neurological deficit, which may coexist with spinal pathology and can lead to persistence or deterioration of symptoms after surgery. Failure to recognize and counsel the patient regarding this pre-existing pathology can lead to unfavorable patient-related outcome measures. Peripheral neuropathy can be diagnosed with electrophysiological studies. Hence, it is recommended to obtain a nerve conduction study during the pre-operative evaluation of suspected diabetic neuropathy cases.

Overdistraction due to a large interbody cage or deformity correction can result in neurological complications through stretching of the nerve roots. One patient in our series had lumbar kyphosis with listhesis, which was corrected while performing TLIF. Immediately after surgery, the patient was found to have foot drop. All obvious causes for the deficit were ruled out. On measuring the distance from the sciatic notch to the L4 foramen on the pre-operative and post-operative radiographs, we found 2.4 cm lengthening postoperatively, which could be a likely cause of the deficit [2], [3].

Other probable causes discussed in the literature include excessive compression over the rod after TLIF, which is a known cause of contralateral radicular symptoms in MIS TLIF. Lumbar decompression leading to iatrogenic pars or facet injury can, in turn, lead to postoperative pain and radiculopathy [13].

There are very few studies in the literature describing immediate postoperative complications in lumbar spine surgery and no study specifically describing NOPD as we have defined it. Most studies on FBSS are concentrated on post-discectomy complications for lumbar disc herniation. The study by Rohde V et al. [4] can be considered similar, where the authors describe immediate failed back surgery syndrome (iFBSS) after lumbar microdiscectomy. The authors found an incidence of 2.8% for iFBSS among 1546 patients studied. All patients with iFBSS underwent reoperation, and the cause was identified in 31 patients. Twenty-two had re-herniation of disc material, 6 patients had epidural hematoma, 2 were found to have inadequate decompression, and one patient was found to have dural tear with fascicle herniation. The authors could not identify any clear cause of iFBSS in 13 patients and labelled them as battered root syndrome. Another notable finding from this study is that the correct diagnosis was identified radiologically in only 57% of the patients.

This study has several important limitations. First, this is a single-center study, meaning the surgical techniques, decision-making, and complication rates reflect the experience of a specific institution, which may limit the broad generalizability of the findings. Second, the sample size of the NOPD cohort (n = 31) is relatively small, precluding meaningful statistical analysis or the identification of significant risk factors. Third, the proposed management algorithm was not formally validated; because the study is purely descriptive and lacks a control group, we cannot definitively assess whether adhering to this algorithm improved clinical outcomes compared with standard clinical judgment. Fourth, the inclusion criteria restrict NOPD to symptoms arising strictly within one month of surgery. While this defines the acute nature of the condition, it limits the applicability of the NOPD concept and algorithm exclusively to the immediate postoperative period. Fifth, several diagnostic labels, particularly battered root syndrome and epidural fibrosis, are highly subjective and serve essentially as diagnoses of exclusion. This subjectivity highlights the difficulty of diagnosing postoperative pain and directly contributes to the fact that the definitive etiology remained unidentified in over half of the cohort, 17 out of 31 patients. Finally, the absence of comprehensive patient-reported outcome measures (PROMs) beyond the VAS and basic neurological grading limits the depth and completeness of the outcomes data. These factors underscore the need for future, well-controlled, multicenter studies to validate the NOPD concept and its diagnostic pathway.

## Conclusions

To our knowledge, this study is among the first to formally introduce and define the specific clinical entity of NOPD. While the conventional terms FBSS and iFBSS are more general and do not differentiate between the persistence of symptoms and the development of new symptoms, the proposed entity, NOPD, is more clearly defined and aims to identify early complications of lumbar spine surgery. We have also proposed a structured, algorithmic approach for the management of NOPD, thereby improving surgical outcomes.

However, in a substantial number of cases, the exact etiology of NOPD remained unidentified despite a structured algorithm. Hence, in conclusion, although better training, technique, and the availability of enabling technology have made spine surgery safer than before, complications beyond a surgeon’s control are inevitable in a certain small percentage of patients.
